# Supplementary material for: Intercomparison of Same-Day Remote Sensing Data for Measuring Winter Cover Crop Biophysical Traits
Source: Sensors (Basel). 2024 Apr 6;24(7):2339. doi: 10.3390/s24072339 (PMC11014063; doi:10.3390/s24072339)
Supplement: Supplementary file 1 [file sensors-24-02339-s001.zip › sensors-2833066-supplementary.pdf]

Supplementary Materials for *Sensors* 2833066

"Intercomparison of same-day remote sensing data for measuring winter cover crop biophysical traits"

Alison Thieme [1,*], Kusuma Prabhakara [2], Jyoti Jennewein [1], Brian T. Lamb [3], Greg W. McCarty [4] and W. Dean Hively [5]

[1] U.S. Department of Agriculture - Agricultural Research Service, Sustainable Agricultural Systems Laboratory, Bldg 001, BARC-W, 10300 Baltimore Avenue, Beltsville, Maryland 20705, United States; alison.thieme@usda.gov; jyoti.jennewein2@usda.gov
[2] Department of Geographical Sciences, University of Maryland, 2181 Samuel J. LeFrak Hall, College Park, Maryland 20742, USA; kusumaprabhak@gmail.com
[3] U.S. Geological Survey, Lower Mississippi-Gulf Water Science Center, Coram, NY 11727, USA; blamb@usgs.gov
[4] U.S. Department of Agriculture - Agricultural Research Service, Hydrology and Remote Sensing Laboratory, Bldg 007, BARC-W, 10300 Baltimore Avenue, Beltsville, Maryland 20705, USA; greg.mccarty@usda.gov
[5] U.S. Geological Survey, Lower Mississippi-Gulf Water Science Center, Beltsville, MD 20705, USA; whively@usgs.gov
* Correspondence: alison.thieme@usda.gov

Disclaimer: Any use of trade, firm, or product names is for descriptive purposes only and does not imply endorsement by the U.S. Government.

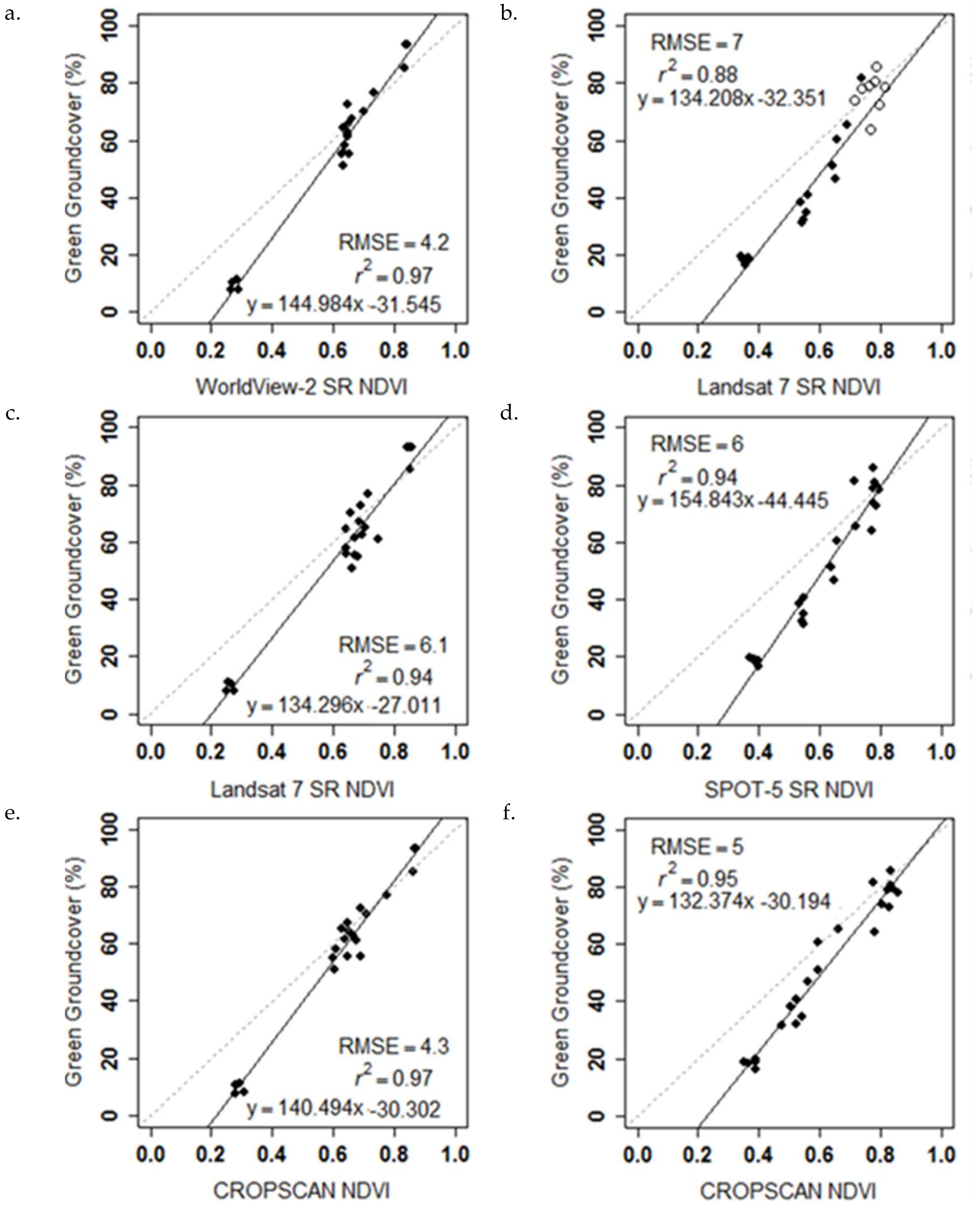

Figure Supplemental 1 (S1). Linear regression of fractional groundcover (%) and NDVI measurements from satellite sensors Landsat 7 (b, c), SPOT 5 (d), and Worldview-2 (a) and proximal sensors CROPSCAN (e, f). Satellite and proximal sensor values were collected on December 6, 2012 (a, c, e) and January 23, 2013 (b, d, f). Fractional groundcover data were derived from RGB photos collected on December 14, 2012 (a, c,

e) and January 23, 2013 (b, d, f) and processed using SamplePoint. The dashed line represents a 1:1 relationship with an intercept of zero. The solid circles are data points that were free of clouds. The hollow circles represent areas that are covered by cloud shadow in the January 23, 2013, Landsat 7 image and were excluded from the linear regression analysis. Linear regression and R^2 values are printed on each panel.

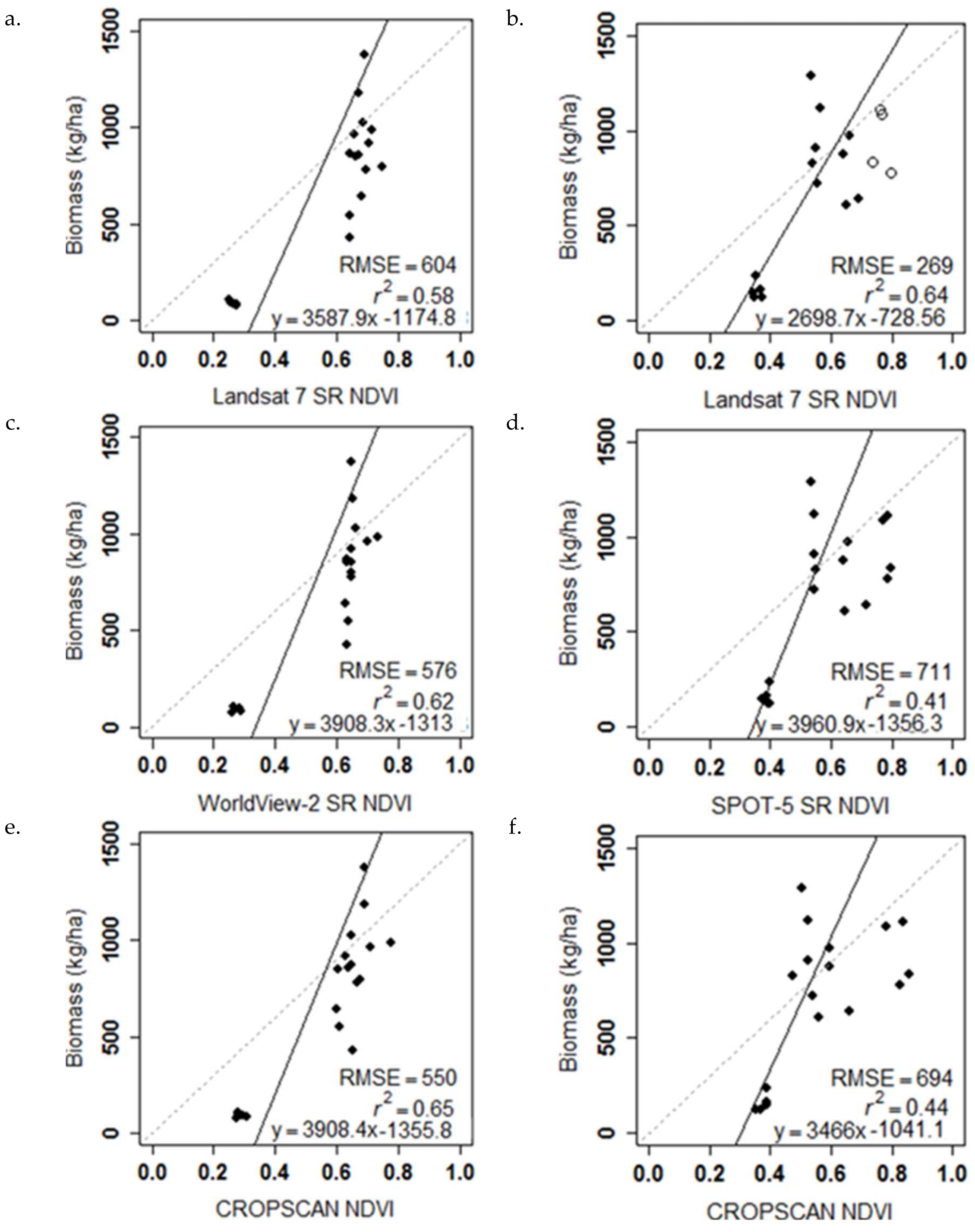

Figure Supplemental 2 (S2). Linear regression of biomass (kg/ha) and NDVI measurements from satellite sensors Landsat 7 (b, c), SPOT 5 (d), and Worldview-2 (a) and proximal sensors CROPSCAN (e, f). Satellite and proximal sensor values were collected on December 6, 2012 (a, c, e) and January 23, 2013 (b, d, f). Biomass data were collected *in situ* collected on December 14, 2012 (a, c, e) and January 10, 2013 (b, d, f). The dashed line represents a 1:1 relationship with an intercept of zero. The solid circles are data points that were free of clouds. The hollow circles represent areas that are covered by cloud shadow in the January 23, 2013, Landsat 7 image and were excluded from the linear regression analysis. Linear regression and R^2 values are printed on each panel.
